# BRCAA1 monoclonal antibody conjugated fluorescent magnetic nanoparticles for *in vivo *targeted magnetofluorescent imaging of gastric cancer

**DOI:** 10.1186/1477-3155-9-23

**Published:** 2011-05-25

**Authors:** Kan Wang, Jing Ruan, Qirong Qian, Hua Song, Chenchen Bao, Xueqing Zhang, Yifei Kong, Chunlei Zhang, Guohan Hu, Jian Ni, Daxiang Cui

**Affiliations:** 1 Department of Bio-nano Science and Engineering, National Key Laboratory of Nano/Micro Fabrication Technology, Key Laboratory for Thin Film and Microfabrication of Ministry of Education, Institute of Micro-Nano Science and Technology, Shanghai Jiao Tong University, 800 Dongchuan Road, Shanghai 200240, China; 2 Department of Surgery, Changzheng Hospital affiliated to Second Military Medical University, 151 Fengyang Road, Shanghai 20003, China

## Abstract

**Background:**

Gastric cancer is 2th most common cancer in China, and is still the second most common cause of cancer-related death in the world. How to recognize early gastric cancer cells is still a great challenge for early diagnosis and therapy of patients with gastric cancer. This study is aimed to develop one kind of multifunctional nanoprobes for *in vivo *targeted magnetofluorescent imaging of gastric cancer.

**Methods:**

BRCAA1 monoclonal antibody was prepared, was used as first antibody to stain 50 pairs of specimens of gastric cancer and control normal gastric mucous tissues, and conjugated with fluorescent magnetic nanoparticles with 50 nm in diameter, the resultant BRCAA1-conjugated fluorescent magnetic nanoprobes were characterized by transmission electron microscopy and photoluminescence spectrometry, as-prepared nanoprobes were incubated with gastric cancer MGC803 cells, and were injected into mice model loaded with gastric cancer of 5 mm in diameter via tail vein, and then were imaged by fluorescence optical imaging and magnetic resonance imaging, their biodistribution was investigated. The tissue slices were observed by fluorescent microscopy, and the important organs such as heart, lung, kidney, brain and liver were analyzed by hematoxylin and eosin (HE) stain method.

**Results:**

BRCAA1 monoclonal antibody was successfully prepared, BRCAA1 protein exhibited over-expression in 64% gastric cancer tissues, no expression in control normal gastric mucous tissues, there exists statistical difference between two groups (*P *< 0.01). The BRCAA1-conjugated fluorescent magnetic nanoprobes exhibit very low-toxicity, lower magnetic intensity and lower fluorescent intensity with peak-blue-shift than pure FMNPs, could be endocytosed by gastric cancer MGC803 cells, could target *in vivo *gastric cancer tissues loaded by mice, and could be used to image gastric cancer tissues by fluorescent imaging and magnetic resonance imaging, and mainly distributed in local gastric cancer tissues within 12 h post-injection. HE stain analysis showed that no obvious damages were observed in important organs.

**Conclusions:**

The high-performance BRCAA1 monoclonal antibody-conjugated fluorescent magnetic nanoparticles can target *in vivo *gastric cancer cells, can be used for simultaneous magnetofluorescent imaging, and may have great potential in applications such as dual-model imaging and local thermal therapy of early gastric cancer in near future.

## Background

Gastric cancer was once the second most common cancer in the word[1]. Up to date, in the United States, stomach malignancy is currently the 14th most common cancer, and 2th most common cancer in China[2,3]. Gastric cancer is still the second most common cause of cancer-related death in the world, and remains difficult to cure because most patients present with advanced disease. Therefore, how to recognize, track or kill early gastric cancer cells is very key for early diagnosis and therapy of patients with gastric cancer.

Up to date, looking for biomarkers closely associated with gastric cancer is still an important task. Since 1998, we have been being tried to establish an early gastric cancer pre-warning system[4], and hope to use this pre-warning system to detect early gastric cancer cells to recognize the patients with early gastric cancer. Although some differently-expressed genes associated with early gastric cancer were identified[5,6], no one gene can be confirmed to be specific biomarker of gastric cancer. Therefore, in order to recognize early gastric cancer cells, we only select potential biomarkers associated with gastric cancer, and combine nanoparticles and molecular imaging techniques, try to find *in vivo *early gastric cancer cells by *in vivo *tumor targeted imaging. In our previous work, we screened out and cloned BRCAA1 gene (breast cancer associated antigen 1 gene) from breast cancer cell line MCF-7cells [AF208045, also called ARID4B (AT-rich interactive domain-containing protein 4B)], and identified its antigen epitope peptide SSKKQKRSHK[7,8]. We also prepared BRCAA1 polyclonal antibody, and observed that the BRCAA1 protein exhibited over-expression in almost 65% clinical specimens of gastric cancer tissues[9-11]. We also observed that BRCAA1 antigen is over-expressed in gastric cancer cell lines such as MKN-1, MKN-74, SGC-7901, KATO-III and MGC803 cells. Therefore, we predict that BRCAA1 protein may be one potential targeting molecule for *in vivo *gastric cancer cells.

In recent years, molecular imaging technologies based on multi-functional nanoprobes have made great progress. For example, nanoparticles such as quantum dots, magnetic nanoparticles and gold nanorods, etc. have been used for molecular imaging[12-19]. So far several small animal imaging technologies have been developed such as optical imaging (OI) of bioluminescence (BLI), fluorescence (FLI) and of intravital microscopy (IVM), micro-PET, MRI and CT[20-26]. Among all these technologies, how to improve their spatial resolution and tissue depth sensitivity is a great challenge. So far *in vivo *tumor tissues with over 1 cm in diameter can be easily identified by CT, MRI, PET and bioluminescence imaging, tumors with less than or equal to 5 mm in diameter is very difficult to be found in clinical patients. In our previous reports, photosensitizer-conjugated magnetic nanoparticles were successfully used for *in vivo *simultaneous magnetofluorescent imaging and targeting therapy[27]. However, the targeting ability of nanoprobes was highly dependent on magnetic nanoparticles. We also prepared a multifunctional Ribonuclease-A-conjugated CdTe quantum dot cluster nanosystem for synchronous cancer imaging and therapy[28], the targeting ability of as-prepared nanoprobes is dependent on RGD peptide. Some studies show that HER-2 protein exhibits abnormal expression in 6-35% gastric cancer tissues[29,30], and has been used as the therapeutic target for clinical patients with gastric cancer[31], therefore, HER-2 protein owns great potential in imaging and therapy of gastric cancer. However, up to date, no report shows that targeted imaging and therapy of *in vivo *gastric cancer is based on biomarkers associated with gastric cancer.

In recent years, we controllably prepared silica-coated quantum dots and super-paramagnetic nanoparticle composites(FMNPs) with strong fluorescent signals and excellent magnetic properties, and have used them for bio-labeling, tracking stem cells, bio-separation, targeting imaging and hyperthermia of tumors[29-32], we also observed that as-prepared nanoparticles own good biocompatibility and stability[33-38].

In this paper, we fully use the advantages of FMNPs and BRCAA1 antigen, prepared monoclonal antibody against BRCAA1 protein, and prepared BRCAA1 monoclonal antibody-conjugated fluorescent magnetic nanoprobes (BRCAA1-FMNPs), employed nude mice model loaded with gastric cancer of 5 mm in diameter and IVIS imaging system and Magnetic Resonance Imaging, investigated the feasibility of as-prepared nanoprobes for non-invasive *in vivo *targeted dual modal imaging of gastric cancer. Results show that as-prepared nanoprobes can be used for *in vivo *dual-model imaging of gastric cancer, and may have great potential in applications such as dual-model imaging and local thermal therapy of early gastric cancer in near future.

## Results and Discussion

### Characterization of anti-BRCAA1 monoclonal Antibody

**Figure 1:**
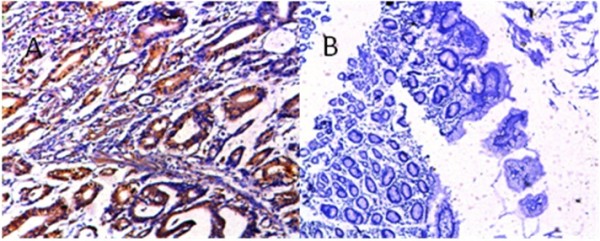
**Expression of BRCAA1 protein in gastric cancer tissues and normal control gastric mucous tissues**. A: gastric cancer tissues, × 100; B: normal control tissues, × 50.

As shown in Table 1, we successfully obtained two positive clone cell lines S-200-5 and S-335-5, their titers were different, finally we selected the anti-BRCAA1 monoclonal antibody from S-200-5 cell line as the first antibody to stain gastric cancer tissues and control tissues. We found that BRCAA1 protein exhibited over-expression in 64% gastric cancer tissues, no expression in normal control gastric mucous tissues, as shown in Figure 1, there exists statistical difference between two group (*P *< 0.01). This result is almost identical to our previous report[4,9-11], which highly suggest that BRCAA1 antigen may be selected as the potential target for most gastric cancer, if as-prepared nanoprobes may recognize 64% patients with early gastric cancer, it will be very useful for diagnosis and therapy of clinical gastric cancer patients.

**Table 1:** Titers of BRCAA1 Monoclonal Antibodies in Ascites Fluid Induced by Hybridoma Clone Cells by ELISA

	Antibody titer*			
	
Clone	BRCAA1 (C)-OVA **	BRCAA1 (C)-BSA **	BSA **	OVA **
**S-200-5**	1,024,000	1,024,000	<1,000	<1,000
**S-335-5**	128,000	512,000	<1,000	<1,000

*The reciprocal of ascites fluid dilution, the first dilution of ascites fluid was 1:1,000.

**The antigens were coated on ELISA plate.

### Preparation and Characterization of BRCAA1- FMNPs nanoprobes

**Figure 2:**
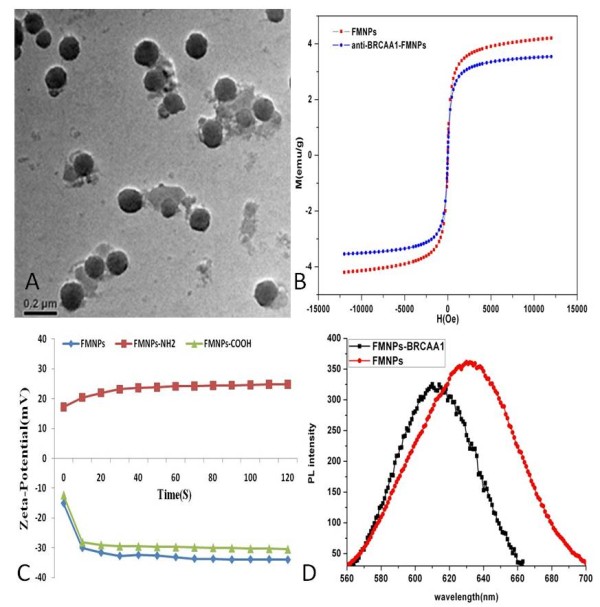
**Characterization of anti-BRCAA1-FMNPs nanoprobes**. A: HR-TEM picture of FMNPs; B: Magnetic property of anti-BRCAA1-FMNPs nanoprobes; C: Zeta-potential of FMNPs with amino group, COOH, Si-O group; D: PL spectra of FMNPs conjugated with and without BRCAA1 antibody.

As shown in Figure 2A, prepared FMNPs were composed of silica-wrapped CdTe and magnetic nanoparticles, their size were 50 nm or so in diameter. As shown in Figure 2D, after FMNPs were conjugated with anti-BRCAA1 antibody, as-prepared nanoprobes' photoluminescence (PL) intensity was lower than that of FMNPs, exhibiting left-shift of 40 nm, which was due to decrease of the polarization rate of the surrounding molecules, and resulting in the decrease of stokes displacement, finally resulting in a blue shift in the emission spectra. Similarly, magnetic intensity of as-prepared nanoprobes was also lower than FMNPs.

In the course of preparing BRCAA1-FMNPs nanoprobes, we found that surface functionalization of FMNPs was very key to conjugate anti-BRCAA1 antibody with FMNPs via covalent bond. As shown in Figure 2C, different functional groups of FMNPs have different zeta-potential values. FMNPs had negative Si-O-group, their zeta-potential value was -34.05 mV, the FMNPs with amino group had positive zeta-potential value of 24.80 mV, FMNPs with carboxyl group had negative zeta-potential value of -30.50 mV. We observed that carboxyl groups on the surface of FMNPs conjugated with anti-BRCAA1 antibody easier than amino groups on the surface of FMNPs. As shown in Table 2, the average coupling rate of anti-BRCAA1 antibody with FMNPs-COOH was 80.28%.

**Table 2:** Coupling rate measurement of FMNPs-anti-BRCAA1 antibody

	Total concentration of the anti-BRCAA1 antibody (ng/μL)	The concentration of anti-BRCAA1 antibody in residual reaction mixture (ng/μL)	Coupling rate (%)
1	1000.0	197.3	80.27

2	1000.0	191.2	80.88

3	1000.0	203.0	79.70

### As-prepared nanoprobes for *in vitro *targeted gastric cancer cells

**Figure 3:**
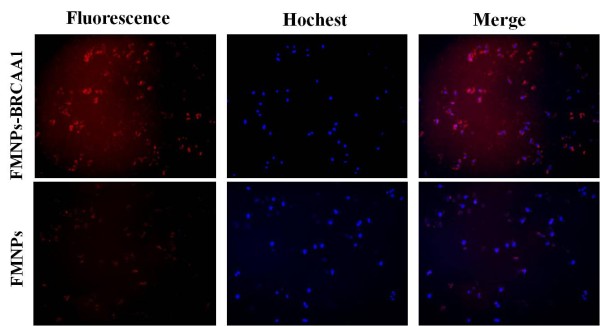
***In vitro *fluorescence images of MGC 803 after treated with FMNPs and FMNPs-BRCAA1 nanoparticles (Magnification= × 200)**. The top group of images illustrated FMNPs random distribute in the cytoplasm, the bottom group of images exhibited FMNPs-BRCAA1 dispersed around the nucleolus and had well targeting ability to the MGC803.

**Figure 4:**
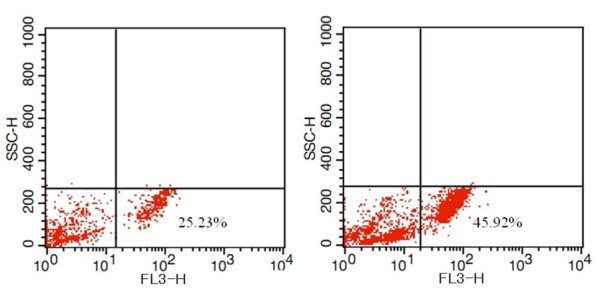
**FACSCalibur Flow cytometer analysis of MGC803 labeled with FMNPs and FMNPs-BRCAA1**. A: the MGC803 treated with 50 μg/mL of FMNPs for 24 h exhibited 25.23% cell were labeled with FMNPs. B: the MGC803 treated with 50 μg/mL of FMNPs-BRCAA1 for 24 h illustrated up to 45.92% cell were labeled with FMNPs-BRCAA1.

**Figure 5:**
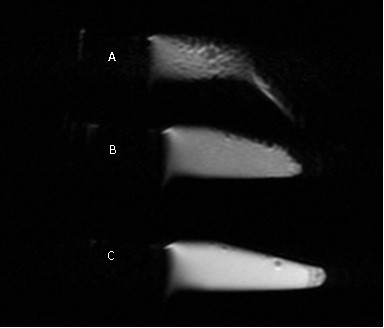
**MR imaging of MGC803 cells and HDF cells**. A: MGC803 cells with anti-BRCAA1-FMNPs. B:HDF cells with anti-BRCAA1-FMNPs. C: MGC803 cells with only FMNPs.

Targeting ability of as-prepared nanoprobes *in vitro *were observed by fluorescence microscope and calculated by FACSCalibur Flow cytometer. As shown in Figure 3, FMNPs randomly dispersed in the inner of the cytoplasm, and anti-BRCAA1-FMNPs nanoprobes existed around the nucleolus. Both FMNPs and prepared BRCAA1-FMNPs nanoprobes can enter into the cytoplasm of MGC803 cells after 4 h incubation with MGC803 cells, as shown in Figure 4A, FMNPs could label 25.23% MGC803 cells, the remain 74.77% cells could not be labeled. As shown in Figure 4B, 45.92% MGC803 cells could be labeled by the BRCAA1-FMNPs nanoprobes. When FMNPs and anti-BRCAA1-FMNPs nanoprobes were respectively incubated with MGC803 cells and human fibroblast cells for 0.5 h, we observed a lot of anti-BRCAA1-FMNPs nanoprobes entered into MGC803 cells, few nanoprobes entered into human fibroblast cells, few FMNPs could enter into MGC803 cells and human fibroblast cells, which highly suggest that anti-BRCAA1-FMNPs nanoprobes can target MGC803 cells specifically. The Magnetic Resonance imaging of MGC803 cells and human fibroblast cells incubated with anti-BRCAA1-FMNPs for 4 h were shown in Figure 5, MGC803 cells exhibited strong magnetic signal than human fibroblast cells (HDF), which also showed that the prepared nanoprobes can target MGC803 cells specifically.

### As-prepared nanoprobes for fluorescent imaging of *in vivo *gastric cancer cells

To evaluate tumor targeted properties of anti-BRCAA1-FMNPs nanoprobes, nude mice models loaded with MGC-803 gastric cancer cells were prepared and monitored under a non-invasive manner for 12 h by using IVIS fluorescence imaging system.

**Figure 6:**
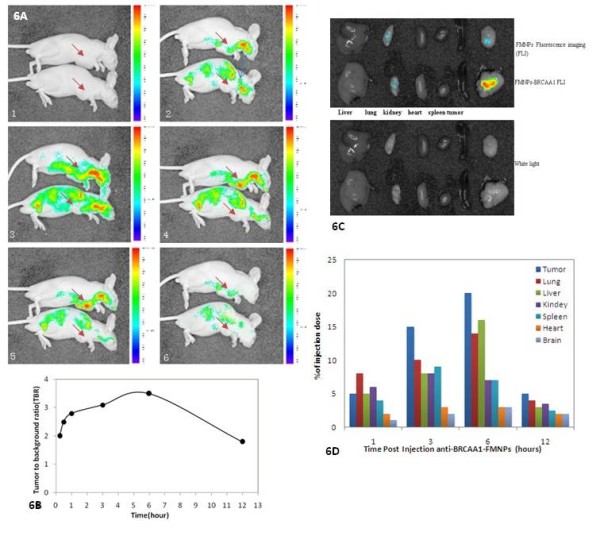
***In vivo *fluorescence images of tumor accumulation and tissue distribution for FMNPs-BRCAA1 nanoparticles in MGC803 human gastric tumor-bearing athymic nude mice**. A, *In vivo *fluorescence images of athymic nude mice bearing. MGC803 human gastric tumor was obtained after injection of FMNPs-BRCAA1 nanoparticles at different time point. The tumor location is specified with an arrow. A-1: 0 h, A-2:0.5 h, A-3:1 h, A-4:3 h, A-5:6 h, A-6:12 h. B, TBR [Tissue to background (muscle) ratio] value. The TBR value was determined as follows: TBR = (Tumor signal-background signal)/(background signal). C, *Ex vivo *fluorescence images of dissected organs and tumor of mice bearing MGC803 human gastric tumor sacrificed at 12 h after injection of FMNPs-BRCAA1 nanoparticles. The fluorescence images of dissected organs and tumor were obtained using a fluorescence imaging technique with a 630 nm emission filter. D, Biodistribution of anti- BRCAA1-FMNPs in mice after intravenous injection. Several time points after injection, iron amounts in tissue samples were evaluated by ICP mass spectrometry (n = 3).

By monitoring real-time fluorescence intensity in the whole body, the tumor-targeting character of the anti-BRCAA1-FMNPs probe was easily determined in the nude mice loaded with gastric cancer MGC803 cells. As shown in Figure 6A, the whole animals produced fluorescent signals within 30 min of post-injection of nanoprobes, the subcutaneous tumor tissues could be clearly delineated from the surrounding background tissue between 1 h and 12 h post-injection, with maximum contrast occurring at 6 h post-injection. Strong fluorescence signal was still be detected in the tumor site at 6 h post-injection, which indicated that the anti-BRCAA1-FMNPs nanoprobes were preferentially accumulated in the tumor tissues. Indeed based on the results in Figure 6B, the higher tumor to background ratio (TBR) value highly suggested that as-prepared nanoprobes preferentially accumulated in tumor tissues compared to normal control tissues. This was confirmed in fluorescence images, which showed that the fluorescence signal of as-prepared nanoprobes in the tumor site was strongest among all mice organs as shown in Figure 6C. In addition, after 12 h post-injection of anti-BRCAA1-FMNPs nanoprobes, fluorescence intensity in tumor was still observed clearly, while the uptake of prepared nanoprobes in normal organs was not obvious. These data highly suggest that prepared nanoprobes can target highly efficiently tumor tissues inside nude mice loaded with gastric cancer. We also observed that those nanoprobes in the whole mouse body almost completely disappeared at 12 h post-injection, we also detected the nanoprobes exited out from the cholecyst system (data not shown), the time-dependent cholecyst clearance of nanoprobes highly suggest that as-prepared nanoprobes can not stay inside nude mice for longer time, thus, as-prepared nanoprobes own good bio-safety.

### Pathological analysis of tumor and important organs

**Figure 7:**
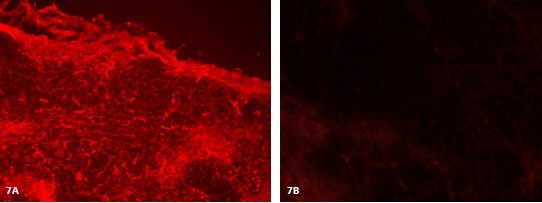
**Result of Immunofluorescence Analysis**. A, tumor tissue. B, liver. (Magnification= × 200).

*In vitro *evaluation of excised major tissues including liver, lung, spleen, kidney, and heart, as well as the tumor, indicated that the anti-BRCAA1-FMNPs probes were mainly up-taken by the tumor tissues, which exhibited strong fluorescence signals, as shown in Figure 7, whereas other tissues including liver, lung, spleen and heart up-took anti-BRCAA1-FMNPs nanoprobes very less, which furtherly indicates that as-prepared BRCAA1-FMNPs nanoprobes can target gastric cancer tissues. We also used HE staining to check all organs, no obvious damages were observed in important organs [see additional file 1].

### As-prepared nanoprobes for MR Imaging of nude mice loaded with gastric cancer

**Figure 8:**
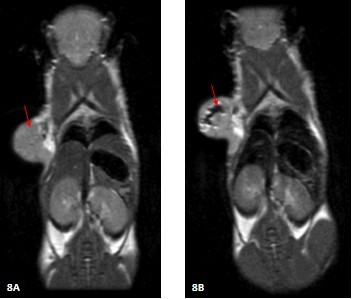
**MRI image of mice**. A, FMNPs without coupling BRCAA1 B, FMNPs coupled with BRCAA1

*In vivo *MR imaging was performed on nude mice loaded with subcutaneous gastric cancer at 12 h post-injection. Representative images of T2 maps were shown in Figure 8, after injecting the nanoprobes, a significant change in signal intensity was observed in some regions of tumors, indicating that there existed accumulation of the nanoprobes in tumor site as shown in Figure 8B, as the arrow showed. As a control, after the mice model with gastric cancer were injected FMNPs for 12 h, the mice were performed MR imaging, which did not show intensive signal in tumor area (Figure 8A).

### Potential mechanism of targeting imaging

In recent years, molecular imaging technologies have been used for real-time and non-invasive imaging of *in vivo *tumor tissues[39-43]. For example, quantum dots, due to their unique photoluminescent properties, have been used for bio-labeling and fluorescent imaging[11-13,33,43], but quantum dots' toxicity limited their application in human body, so far some safe quantum dots are being developed. Magnetic nanoparticles have also been used as contrast reagent for MR imaging[15,33,36]. At the same time, combination of two imaging modalities provides the advantages of both than using one method, which would provide comprehensive information on tumor localization, environment, and status.

In this study, we designed and prepared a novel imaging probe, which was composed of silicon-wrapped quantum dots and magnetic nanoparticles with the aim of enhancing their biocompatibility. Our results show that prepared silicon-wrapped quantum dots and magnetic nanoparticles are very stable, and own strong fluorescent signals and magnetic intensity. Using the strong fluorescent signals of as-prepared nanoprobes, we successfully obtained the fluorescent images of *in vivo *gastric cancer tissues with 5 mm in diameter in nude mice model. Using the strong magnetic signals of as-prepared nanoprobes, we also successfully obtained MR images of *in vivo *gastric cancer tissues with 5 mm in diameter in nude mice model. Compared with previous reports, bigger size of tumor tissues (>5 mm ) could be easily imaged by using fluorescent imaging and MRI imaging, as a contrast, our results showed that as-prepared nanoprobes can detect smaller size of tumor tissues (less 5 mm in diameter), which markedly improved the sensitivity of detection method. Our result also is the first time to report dual-modal targeting imaging of *in vivo *gastric cancer tissues.

How to target *in vivo *gastric cancer tissues is also a challengeable problem. Up to date, no specific gastric cancer biomarkers were reported. Although HER-2 protein was confirmed to have positive expression in 6-35% of gastric cancer tissues[28-31], HER-2 protein also exhibits over-expression in many tumor tissues such as breast cancer, lung cancer, colon cancer, etc, therefore HER-2 should not be specific biomarker for gastric cancer. Our results showed that BRCAA1 antigen is only over-expressed in 64% or so of gastric cancer tissues from clinical surgery patients, we also confirmed that BRCAA1 antigen is over-expressed in some gastric cancer cell lines such as MKN-1, MKN-74, SGC-7901, KATO-III and MGC803[6-9]. We used MGC803 cells to prepare nude mice model loaded with gastric cancer, and successfully observed that as-prepared nanoprobes preferentially accumulated in tumor tissues compared with normal control tissues, and as the post-injection time increased. We also observed that injected nanoprobes in the whole body exhibited the time-dependent clearance and the fluorescent signals gradually decreased as the time elapsed due to the liver-cholecyst excretion system and kidney clearness of as-prepared nanoprobes. Several reports showed that kidney only clear nanoparticles with 5 nm in diameter, in our study, we observed that as-prepared nanoprobes with 50 nm in diameter also could be cleared within 12 h. This concrete mechanism is under way.

Nanoprobe biosafety is also an important problem[44], which decides the application prospect of as-prepared nanoprobes. Our results fully showed that as-prepared nanoprobes did not damage important organs including liver, kidney, heart, lung, etc, also did not exhibit long-term staying in important organs, which highly suggest that as-prepared nanoprobes own good biocompatibility, and have great potential in applications such as dual model imaging and selective therapy of early gastric cancer.

## Conclusion

We successfully prepared a novel anti-BRCAA1-FMNPs nanoprobes, which can be used for *in vivo *two modal imaging such as fluorescent imaging and magnetic resonance imaging, and own an obviously specific targeting ability toward a gastric cancer tissues with 5 mm in diameter during 0.5 h and 12 h of post-injection, and own good biocompatibility. This should be first report. The as-prepared multifunctional nanoprobes also can be used for hyperthermia therapy of gastric cancer under *in vitro *alternating magnetic field irradiation, and have great potential in applications such as simultaneous imaging and targeting therapy of clinical gastric cancer in near future.

## Materials and methods

### Preparation of anti-BRCAA1 monoclonal Antibodies

Animal experiments were performed according to Guidelines for Animal Care and Use Committee, Shanghai Jiao Tong University. Monoclonal antibodies were prepared against a purified fusion protein BRCAA1. BALB/c female mice, 4-6 weeks old, were purchased from the Shanghai LAC Laboratory Animal Co. Ltd., Chinese Academy of Sciences (Shanghai, China). The mice were immunized by intraperitoneal injection with 50 μg of purified BRCAA1 protein which was emulsified with an equal volume of Freund's complete adjuvant. Three further injections were administered using incomplete adjuvant every two weeks. Three days after the last injection, the spleen cells of the mice were harvested and fused with the Sp 2/0 mouse myeloma cell line. After 10-14 days, the culture supernatants were screened with an ELISA test in which the solid phase was coated with the recombinant BRCAA1 protein (2 μg/mL) used for the immunization. In the screening process, the monoclonal antibodies to bind with coated BRCAA1 protein were selected. By twice limiting dilution, positive colonies were subcloned. Ascitic fluids were harvested from the mice primed with a 0.5 mL intraperitoneal injection of Pristane and then injected with 10^6 ^hybridoma cells. The class and subclass of each mAb were determined using a mouse monoclonal antibody isotyping kit (Hy Cult Biotechnology B.V., Netherlands). The mAbs were purified from the mouse ascetic fluids using a protein G-Sepharose 4FF (Pharmacia, Uppsala, Sweden) column according to the manufacturer's instructions to remove components which might interfere with the biopanning experiments. The antibody titers were determined by ELISA methods[45]. Finally one of prepared anti-BRCAA1 monoclonal antibodies was used as first antibody to stain 50 specimens of gastric cancer and control gastric mucous tissues, which were collected from Changzheng Hospital and No.1 People Hospital in Shanghai and identified by pathological examination.

### Preparation and Surface Functionalization of FMNPs

Preparation of Fe_3_O_4 _nanoparticles was based on co-precipitation of ferrous and ferric ion solutions (1:2 molar ratio)[46-49]. CdTe nanocrystals were synthesized as follows according to our previous report: CdCl_2 _(5 mmol) was dissolved in 110 ml of water, and 12 mmol of TGA were added under stirring, followed by adjusting the pH to 11 by dropwise addition of 1 M NaOH solution. The mixed solution was placed in a three-necked flask deaerated by N_2 _bubbling for 30 min. Under stirring, 2.5 mmol of oxygen-free NaHTe solution was injected into the three-necked flask, which was freshly prepared from tellurium powder and NaBH_4 _(molar rate of 1:2) in water at 0°C. The resulting solution was about 4 mg/ml, and the 3.5 nm diameter product emitted with a maximum around 630 nm. Fluorescent magnetic nanoparticles (FMNPs) were prepared using the reverse microemulsion approach. Before coupling the FMNPs with the BRCAA1, we first functionalized the surface functional group of FMNPs as carboxyl group. 95 mL ethanol and 2 mL 3-Aminopropyltriethoxysilane (APS) were added to form a mixed solution and allowed to react at room temperature for 24 h. The aminosilane-modified FMNPs were separated by permanent magnet and were washed with deionized water three times. Then redispersed the FMNPs-NH_2 _in 100 mL Dimethylformamide (DMF), added excessive succinic anhydride to form a mixed solution and react at room temperature for 24 h. The carboxyl-modified FMNPs were separated by permanent magnet again and washed with deionized water three times.

### Preparation and characterization of BRCAA1 antibody-conjugated FMNPs

We used two-step process to obtain stable anti-BRCAA1-FMNPs conjugation[48,49]. 1.5 mg FMNPs-COOH solution was dispersed in 2 mL pH7 PBS buffer, and was sonicated for 10 min. Then we mixed 1 mL of fresh 400 mM EDC and 100 mM NHSS in pH 6.0 MES buffer and rotated it at room temperature for 15 min. After this, the resulting solution was separated by magnetic field and 1 mg/mL BRCAA1 monoclonal antibody were added to the above mixture, stirred in dark place for 2 h. To remove free BRCAA1, the residual reaction mixture was separated by magnetic field and the solid remaining was washed with 1 mL PBS buffer three times. Finally, 1 mL 0.05% Tween-20/PBS was added to the BRCAA1-FMNPs conjugation and the final bio-conjugation was stored at 4°C. When we used, this BRCAA1-FMNPs conjugation should be diluted with PBS/0.05% Tween-20. Then we used the Nano Drop device to quantify the coupling rate of BRCAA1 antibody with FMNPs-COOH. Before coupling reaction, we measured the total concentration of BRCAA1 antibody. After coupling reaction, we measured the BRCAA1 antibody concentration in residual reaction mixture and calculated the coupling rate according the equation:

Coupling (%) = (1-Concentration of BRCAA1 antibody in residual reaction mixture/Total concentration of BRCAA1 antibody) × 100.

The as-prepared nanoprobes and pure FMNPs were characterized by transmission electron microscopy and photoluminescence (PL) spectrometry, and Zeta potential analyzer.

### Nanoprobes for *in Vitro *targeting imaging of gastric cancer cells

Gastric cancer cell line MGC803 cells with over-expressed BRCAA1 protein were used as target cells, human fibroblast cells without expressed BRCAA1 protein was used as control, were cultured and collected, and then were treated with 50 μg/mL BRCAA1-FMNPs nanoprobes and cultured in a humidified 5% CO_2 _balanced air incubator at 37°C for 4 h, meanwhile the MGC803 and human fibroblast cells were treated with FMNPs as the control group. Afterward, the cells were rinsed with PBS three times, and then fixed cells with 2.5% glutaraldehyde solution for 30 min. For nuclear counterstaining, MGC803 were incubated with 1 mM Hoechst 33258 in PBS for 5 min. The cells were observed by fluorescence microscope (NIKON TS100-F), and imaged by GE HDX 3.0T MR imaging instrument equipped with ParaVision 3.0 software.

We also used the Flow cytometer to evaluate the gastric cancer cell targeting ability of BRCAA1-FMNPs nanoprobes. MGC803 cells were treated with 50 μg/mL BRCAA1-FMNPs or FMNPs and harvested after 4 h, and then we fixed the cells with 70% ethanol/PBS for 30 min on ice. Approximately 4 × 10^5 ^cells were centrifuged and resuspended with PBS, which were kept on ice until analysis. The number of cells which have been labeled with BRCAA1-FMNPs conjugation or FMNPs were analyzed by BD FACS Calibur Flow cytometer.

### Nanoprobes for fluorescence imaging of nude mode loaded with gastric cancer

Animal experiments were performed according to Guidelines for Animal Care and Use Committee, Shanghai Jiao Tong University. Male athymic nude mice were obtained from Shanghai LAC Laboratory Animal Co. Ltd., Chinese Academy of Sciences (Shanghai, China). MGC-803 cells (1 × 10^6^) were injected subcutaneously into the right anterior flank area of male nude mice with 4 to 5 weeks ages. Tumors were allowed to grow to a diameter of approximately 5 mm. At that point, about 40 μg BRCAA1-FMNPs nanoprobes was injected into the mice (*n *= 3) via the tail vein. Mice were respectively monitored in a non-invasive manner at 0.5, 1, 3, 6, and 12 h to get fluorescence images. Then, tumor and major organs were collected, and were placed on black papers, and subjected to IVIS Lumina imaging system (Xenogen) with emission wavelengths of 630 nm. The fluorescence images[33] were acquired and total fluorescence flux for each sample was obtained. For the control experiment, mice (*n *= 3) were injected via tail vein with 40 μg of FMNPs and subjected to optical imaging at various time points post-injection. Identical illumination settings (e.g., lamp voltage, filter, exposure time) were used in all animal imaging experiments.

### Nanoprobes for MR imaging of nude mice loaded with Gastric Cancer

For MR imaging[33], gastric MGC-803 cells (1 × 10^6^) were injected subcutaneously into the right anterior flank area of male nude mice (*n *= 3) with 4 to 5 weeks ages. After tumors reached approximately 5 mm in diameter, mice were injected with the BRCAA1-FMNPs nanoprobes. MR imaging was performed within 12 h after injections on animals anesthetized with 0.4% pentobarbital. MR imaging was performed using 3.0T field intensity by GE HDX 3.0T MR imaging instrument equipped with GE Signa Excite 3.0T MRI software. The imaging protocol consisted of coronal and transverse T2- weighted spin echo (SE) pulse sequences. To produce T2 maps, the following imaging parameters were used: TR/TE = 1000/10, 20, 30, 40, 50, 60, 70, 80 ms; FOV= 8.0 cm; NEX = 2; slice thickness= 2.0 mm. The mice (*n *= 3) model with gastric tumor performed MR imaging and injected FMNPs without labeling BRCAA1 were used for the negative control. Representative T2 maps of the animals loaded with tumors treated with FMNPs and BRCAA1-FMNPs, respectively. Coronal images showed a significant signal in BRCAA1-over-expressed tumors within 12 h after administration of the BRCAA1-FMNPs nanoprobes.

### Fluorescence microscopy observation and immunofluorescence analysis

To compare the distributions of as-prepared nanoprobes in tissue and tumor, the mice in test group were euthanized after *in vivo *imaging. For histological evaluation, excised tumor and important organs were frozen and embedded by medium at -20°C, and then were sectioned into 8 μm slices, which were used for fluorescence examination under inverted fluorescence microscope (Olympus IX71) equipped with digital camera and immunohistochemical study with BRCAA1 antibody. Digital images were processed with self-software (Image-Pro Plus Version6.3). The important organ slices from heart, lung, kidney, brain and liver were analyzed by hematoxylin and eosin(HE) stain method.

### Statistical Analysis

Each experiment was repeated three times in duplicate. The results were presented as mean ± SD. Statistical differences were evaluated using the *t-test *and considered significance at *P *< 0.05.

## Competing interests

The authors declare that they have no competing interests.

## Authors' contributions

KW and JR carried out the molecular genetic studies, KW and ZX participated in the sequence alignment and drafted the manuscript. CCB and HS carried out the immunoassays. DXC and JN participated in the design of the study and performed the statistical analysis. QRQ and GHH helped to collect clinical specimens from patients with gastric cancer, CLZ collected the normal control tissues, YFK prepared the FMNPs. All authors read and approved the final manuscript.

## Supplementary Material

Additional file 1 The results of important organs stained by HE A: heart; B:liver; C:spleen; D:lung; E:kidney; F: brain Click here for file
