# Supplementary figures and images for: BRCAA1 monoclonal antibody conjugated fluorescent magnetic nanoparticles for in vivo targeted magnetofluorescent imaging of gastric cancer
Source: J Nanobiotechnology. 2011 May 25;9:23. doi: 10.1186/1477-3155-9-23 (PMC3127991; doi:10.1186/1477-3155-9-23)

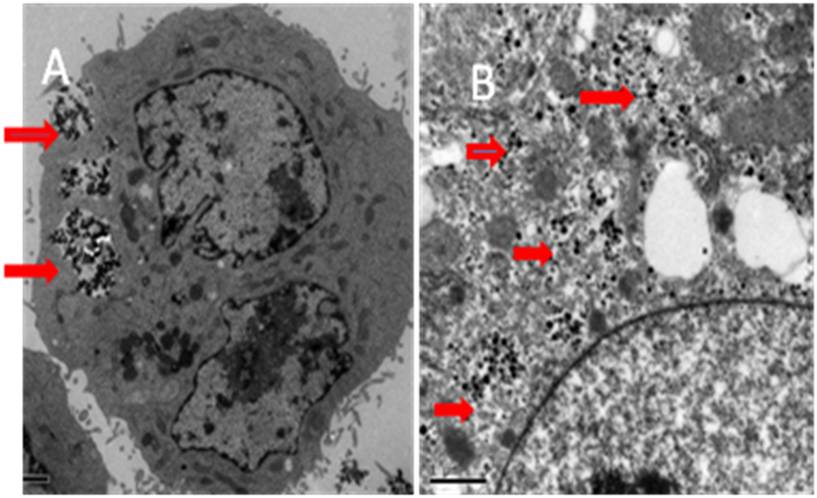

Supplement: Additional file 1 — The results of important organs stained by HE A: heart; B:liver; C:spleen; D:lung; E:kidney; F: brain [file 1477-3155-9-23-S1.JPEG]
